# Anticancer Effects of Resveratrol-Loaded Solid Lipid Nanoparticles on Human Breast Cancer Cells

**DOI:** 10.3390/molecules22111814

**Published:** 2017-10-25

**Authors:** Wenrui Wang, Lingyu Zhang, Tiantian Chen, Wen Guo, Xunxia Bao, Dandan Wang, Baihui Ren, Haifeng Wang, Yu Li, Yueyue Wang, Sulian Chen, Baoding Tang, Qingling Yang, Changjie Chen

**Affiliations:** 1 Department of Biotechnology, Bengbu Medical College, Anhui, Bengbu 233030, China; wenrui-wang1983@163.com (W.W.); 18895670931@163.com (W.G.); baozi7586@163.com (X.B.); dd18895681360@163.com (D.W.); 18895689061@163.com (B.R.); 2 Clinical Testing and Diagnose Experimental Center, Bengbu Medical College, Anhui, Bengbu 233030, China; qyqx1020153528@163.com (L.Z.); ttctoyy@gmail.com (T.C.); 18297318021@163.com (H.W.); liyu1234july@163.com (Y.L.); 18755295203@163.com (Y.W.); 3 Department of Biochemistry and Molecular Biology, Bengbu Medical College, Anhui, Bengbu 233030, China; csl9557@sina.com; 4 Department of Oncology, Bengbu Medical College, Anhui, Bengbu 233030, China; baodtang_16@163.com

**Keywords:** solid lipid nanoparticle, resveratrol, breast cancer, cyclinD1

## Abstract

In this study, resveratrol-loaded solid lipid nanoparticles (Res-SLNs) were successfully designed to treat MDA-MB-231 cells. The Res-SLNs were prepared using emulsification and low-temperature solidification method. The Res-SLNs were spherical, with small size, negative charge, and narrow size distribution. Compared with free resveratrol, the Res-SLNs displayed a superior ability in inhibiting the proliferation of MDA-MB-231 cells. In addition, Res-SLNs exhibited much stronger inhibitory effects on the invasion and migration of MDA-MB-231 cells. Western blot analysis revealed that Res-SLNs could promote the ratio of Bax/Bcl-2 but decreased the expression of cyclinD1 and c-Myc. These results indicate that the Res-SLN may have great potential for breast cancer treatment.

## 1. Introduction

Breast cancer is the most common malignant tumor among women worldwide. The main carcinogenic factors—including genetic mutations, endocrine disorders, and decline in immune function—may increase the risk of developing breast cancer [1,2]. Although early diagnosis of the disease has become possible due to advanced detection techniques, the mortality among women suffering from breast cancer has increased. Traditional disease treatments which include chemotherapy, surgery, and radiotherapy are some of the treatment options in breast cancer [3,4]. However, none are effective in controlling breast cancer, especially in patients with an advanced stage of the disease. Therefore, it is critical to develop more effective strategies for breast cancer.

**Figure 1:**
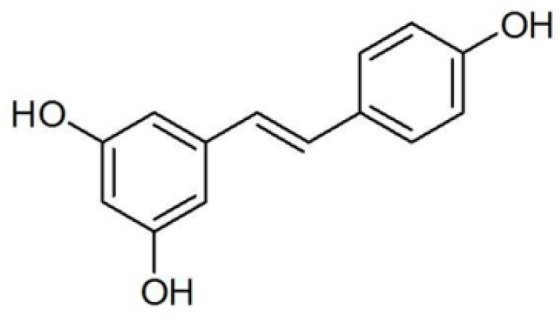
The chemical structure of Resveratrol (Res).

Resveratrol (trans-3,5,4′-trihydroxystilbene,Res) is a natural substance which contains a stilbene class structure of flavonoid polyphenol compounds (Figure 1) [5]. In many studies, resveratrol has shown various biological activities, such as anti-inflammatory, anti-oxidant, anti-obesity, and anti-bacterial activity [6,7,8]. In the past few years, it has also been found to have potential antitumor activity to some cancers, such as ovarian, breast, and prostate cancer [9,10,11]. However, its pharmacokinetic properties are less favorable, since resveratrol has poor aqueous solubility. Moreover, the metabolism of resveratrol is extremely rapid and extensive, and its plasma half-life is very short [12]. In order to solve these problems, it is necessary to find a reasonable strategy to overcome some of the above-mentioned drawbacks and increase its application in cancer treatment.

The development of drug delivery system in cancer treatment has attracted many researchers interest in recent years. Solid lipid nanoparticles (SLNs) have been under consideration for drug delivery system because they possess good physicochemical properties, have the possibility of modulating drug release, and can be metabolized by a variety of organisms. In addition, the SLNs can better protect the encapsulated drugs in the biological circumstances and offer better physiochemical features [13]. In our previous study, we successfully loaded curcumin into SLNs for asthma treatment [14].

Herein, the objective of this study was to design Res-loaded SLNs (Res-SLNs) for breast cancer treatment. Particle size, entrapment efficiency, zeta potential, UV–vis spectra and XRD pattern of the Res-SLNs were investigated. Furthermore, the anti-cancer effect of Res-SLNs was evaluated on MDA-MB-231 human breast cancer cells.

## 2. Results

### 2.1. Morphology, Size Distribution, Zeta Potential, and Drug Loading of Res-SLNs 

Res-SLNs were prepared using emulsification and low-temperature solidification methods.

**Figure 2:**
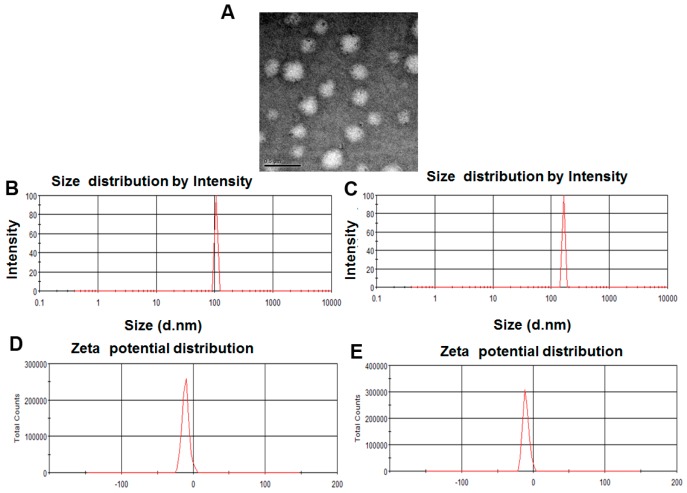
(**A**) Morphology of samples observed by transmission electron microscopy (TEM), Res-SLNs, Scale bar, 500 nm; (**B**,**C**) The size distribution for SLNs and Res-SLNs; (**D**,**E**) The zeta potential for SLNs and Res-SLNs.

The shape and morphology of resveratrol-loaded SLNs were observed by transmission electronic microscopy (TEM). As shown in Figure 2A, the images illustrates that the Res-SLNs are solid spherical particles without aggregation. The mean particle size of SLNs is about 116 nm ± 8.2 nm (PDI = 0.35 ± 0.05) (Figure 2B), Res-SLNs is about 168 ± 10.7 nm with a narrow polydispersity index (PDI = 0.26 ± 0.03) (Figure 2C). Figure 2D,E shows that the zeta potential of the SLNs and Res-SLNs is −22.6 ± 0.8 mV and −23.5 ± 1.6 mV, respectively. The drug-loading capacity is calculated as 25.2 ± 1.7%.

### 2.2. UV–Vis and Powder X-ray Diffraction of Res-SLNs

**Figure 3:**
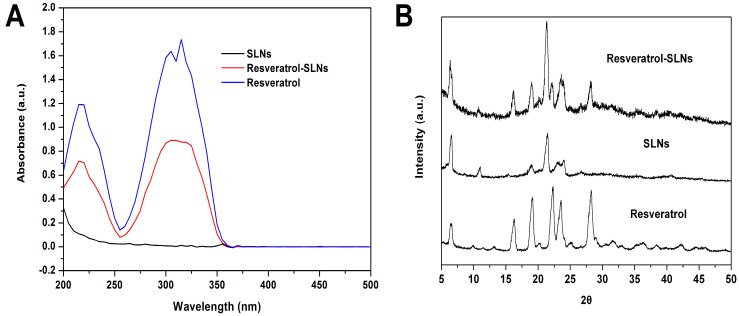
(**A**) UV–vis curves of Resveratrol, SLNs, Resveratrol-SLNs; (**B**) X-ray diffraction curves of Resveratrol and Resveratrol-SLNs.

UV–vis was taken to confirm the presence of resveratrol in Res-SLNs. As shown in Figure 3A, Res and Res-SLNs both show the typical absorbance peaks of Res at 304 nm, which indicates the presence of resveratrol in solid lipid nanoparticles.

The X-ray diffraction technique was employed to confirm the XRD pattern of Res, SLNs, and Res-SLNs. As shown in Figure 3B, the diffraction pattern of Res shows different peaks at 2θ value of 16.36°, 19.18°, 22.67°, 23.02°, 27.67°, indicating highly crystalline structures. As for the XRD pattern of Res-SLNs, similar peaks are also observed, which demonstrates that Res was successfully encapsulated into SLNs.

### 2.3. In Vitro Cytotoxicity 

**Figure 4:**
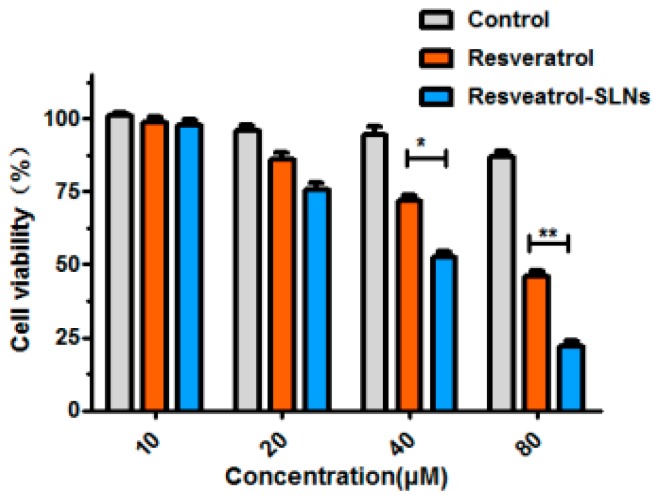
Cell viability of MDA-MB-231 cells measured by using the SRB assay after exposure to free Resveratrol and Resveratrol-SLNs (*n* = 3). Bars represent mean ± S.D. and asterisks denote a significant difference (* *P* < 0.05; ** *P* < 0.01 and *** *P* < 0.001). Data are representative of at least three independent experiments

The cytotoxicity of resveratrol and Res-SLNs against MDA-MB-231 breast cancer cell was investigated by SRB assay and the half inhibition concentration (IC_50_) was calculated. At various concentrations, as presented in Figure 4, SLNs did not show an obvious toxicity to MDA-MB-231 cells after 24 h incubation because of the physiological and biocompatible materials utilized in the synthesizing process. Our results also demonstrated that free Res and Res-SLNs inhibited cell viability in a dose-dependent manner. The IC_50_ value was estimated at 40.82 ± 3.92 μg/mL for Res-SLNs-treated group and 72.06 ± 7.85 μg/mL for resveratrol-treated group, respectively. It is evident that Res-SLNs exhibited obviously more effective cell cytotoxicity in comparison with free resveratrol.

### 2.4. Effect of Res-SLNs on the Apoptosis and Cell Cycle of Breast Cancer Cells 

**Figure 5:**
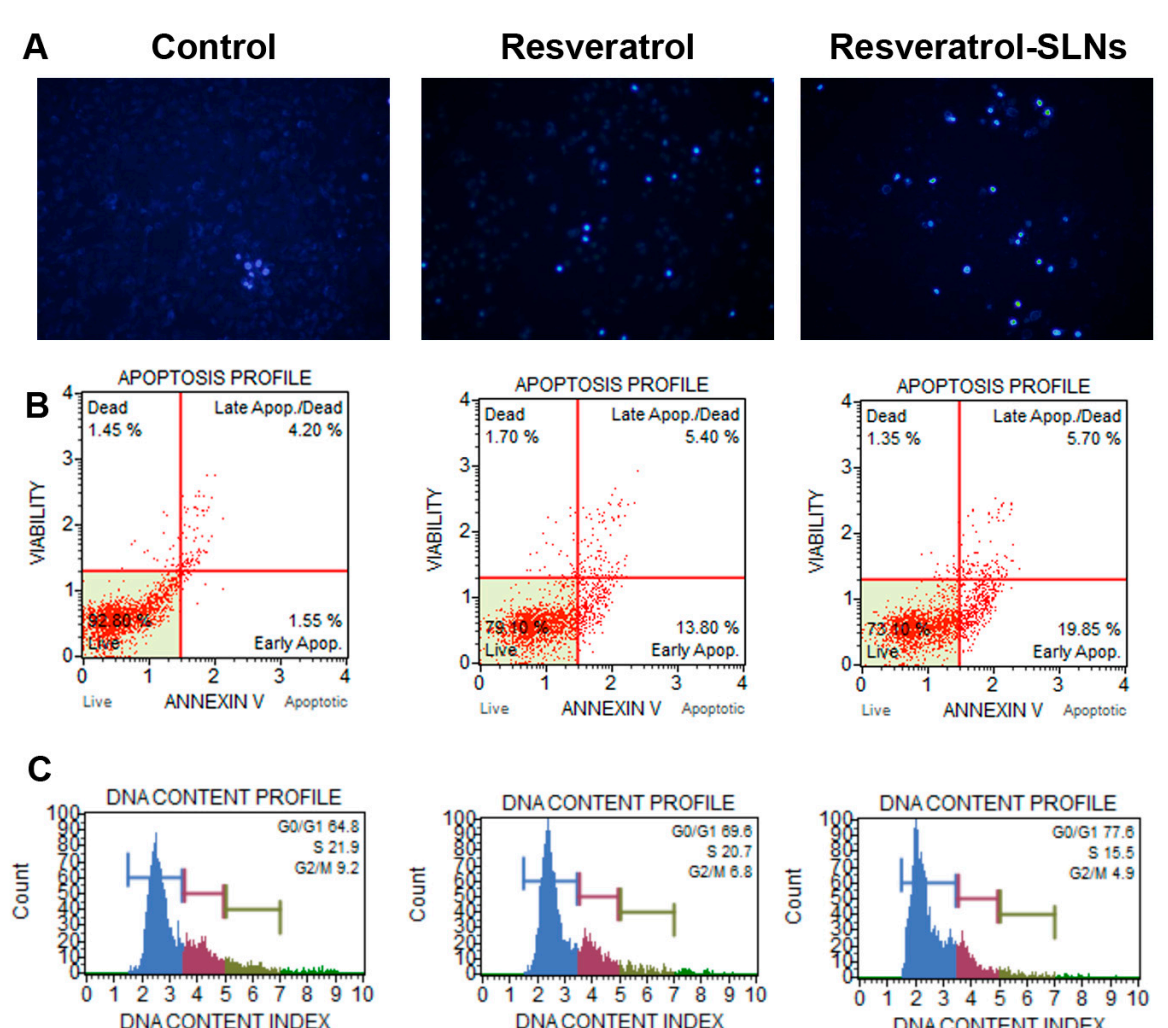
(**A**) MDA-MB-231 cells (×100) stained by Hoechst 33342 after treatments with Resveratrol and Resveratrol-SLNs for 24 h; (**B**) Apoptosis analyzed in MDA-MB-231 cells after 24 h treatment with Resveratrol and Resveratrol-SLNs; (**C**) Cell cycle of MDA-MB-231 cells analyzed 24 h after the treatment of Resveratrol and Resveratrol-SLNs, respectively.

Hoechst staining was used to measure cell apoptosis. The presence of apoptotic nuclei was demonstrated by staining with Hoechst 33342 in MDA-MB-231 cells. As shown in Figure 5A, nuclei shrinkage and fracture appeared after Res-SLNs treatment, suggesting that Res-SLNs caused apoptosis in breast cancer cells. Next, the rate of apoptosis and the distribution of cell cycle phases in different groups were determined by flow cytometric analysis. Only 5.75% apoptotic cells were observed in control group whereas the percentage of apoptotic cells increased up to 19.2% and 25.6% in the free Res-treated group and Res-SLNs-treated group, respectively. The results indicated that Res-SLNs exhibited more potential to induce cell apoptosis. Furthermore, the effects of Res and Res-SLNs (40 µM) on cell cycle progression were investigated in MDA-MB-231 breast cancer cell. As shown in Figure 5B, compared to control group, the percentage of cells in the G0/G1 phase both increased in the free resveratrol and Res-SLNs-treated groups. However, Res-SLNs caused a higher percentage of G0/G1 cell cycle arrest (25.5 ± 1.38%) than free resveratrol (19.2 ± 0.68%) in the same conditions. These results indicated that Res-SLNs were more effective than free drug in inducing cell cycle G0/G1 arrest in MDA-MB-231 cells.

### 2.5. Effect of Res-SLNs on MDA-MB-231 Migration and Invasion

**Figure 6:**
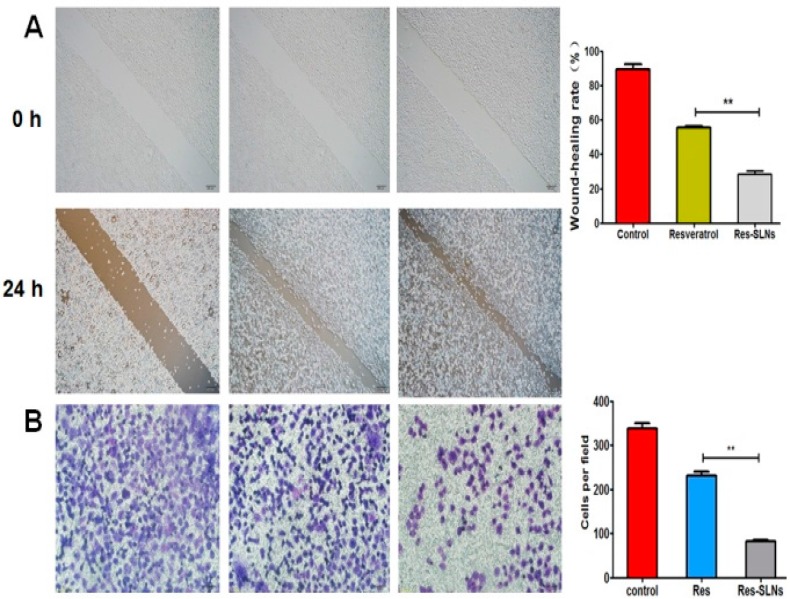
The suppression of MDA-MB-231 cell migration and invasion in vitro by Resveratrol-SLNs. (**A**) The effect of Resveratrol and Resveratrol-SLNs on cell migration measured by wound healing assay; (**B**) Cell invasiveness measured by Matrigel transwell invasion assays after incubation with Resveratrol and Resveratrol-SLNs for 24 h. Bars represent mean ± S.D. and asterisks denote a significant difference (** *P* < 0.01).

The effects of resveratrol and res-SLNs on cell migration in MDA-MB-231 cells were determined by a scratch wound method. As shown in Figure 6A, compared with resveratrol, res-SLNs markedly inhibited MDA-MB-231 cell migration. We further evaluated the inhibition effect of resveratrol and res-SLNs on cell invasion. As shown in Figure 6B, res-SLNs exhibited the greater inhibition effect on tumor cell invasion compared to free resveratrol. This result was consistent with the result of cell migration. 

### 2.6. Effect of Res-SLNs on the Expression of Bcl-2 Family Members and Cell Cycle Regulatory Proteins in MDA-MB-231 Cells

**Figure 7:**
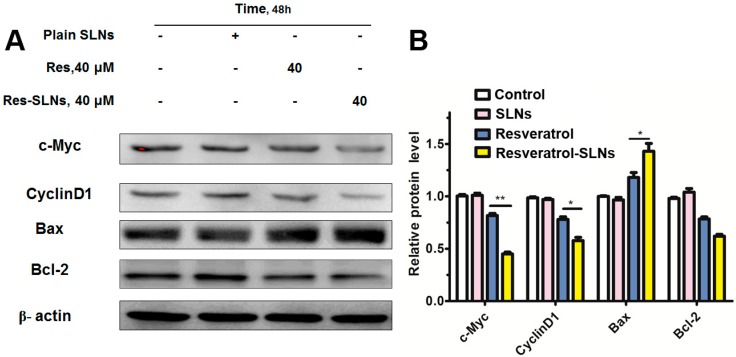
(**A**) c-Myc, Cyclin D1, Bax and Bcl-2 protein expression was analyzed by western blotting in MDA-MB-231 cancer cells after 48 h of treatment with Resveratrol or Resveratrol-SLNs, β-actin was used as a loading control; (**B**) Densitometric quantification of three independent experiments (means ± SD) was performed using Image J. Asterisks denote a significant difference (* *P* < 0.05,** *P* < 0.01).

Since Bcl-2 family members play a major role in apoptosis, the expression of Bax and Bcl-2 in MDA-MB-231 cells were analyzed by the treatment of SLNs, resveratrol and Res-SLNs. As presented in Figure 7A, there was a slight downregulation of Bcl-2 expression in the resveratrol group, while the expression level of Bcl-2 in the res-SLNs group was significantly changed. Compared with the resveratrol group, the Bax expression level in the Res-SLNs group was upregulated (*p* < 0.05) and the Bcl-2/Bax ratio was decreased. Conclusively, the res-SLNs exhibited a more effective ability than free resveratrol in inducing cell apoptosis, probably because the water solubility property of resveratrol was increased when it incorporated into SLNs.

Next, the effects of resveratrol and res-SLNs on the expression of c-Myc and cyclin D1 in MDA-MB-231 cell were examined by Western blotting. Recent studies have demonstrated that cyclin D1 and c-Myc were important target genes of the Wnt signaling pathway. c-Myc acts as an oncogenic transcription factor. The elevation of c-Myc level markedly increases both metastasis and invasion capabilities of tumors. Cyclin D1 regulates cell cycle by controlling G1/S transition, and the upregulation of cyclin D1 contributes to multiple cancers. As shown in Figure 7A, treatment of resveratrol alone resulted in the inhibition of cyclinD1 and c-Myc expression. By comparison, res-SLNs were more effective in inhibiting the expression of cyclinD1 (*p* < 0.05) and c-Myc (*p* < 0.01). These data suggested that res-SLNs could regulate the cell cycle by inhibiting the expression of cyclin D1 and c-Myc.

## 3. Discussions

Nanomedicine provides an excellent platform for drug delivery of anticancer agents in order to enhance their targeting ability and bioavailability. Some of the potential drug delivery systems are micelles, liposomes, polymeric nanoparticles, and solid lipid nanoparticles (SLNs) [15,16,17,18].

In this study, resveratrol was successfully incorporated into SLNs. Res-SLNs was developed using an emulsification and low-temperature solidification method. Most of the materials for preparing SLNs are low cost. The lipids and surfactants are the two main materials used in SLNs. Stearic acid, a saturated monoacid triglyceride, was used as a negatively charged solid lipid, and Myrj52 as negatively charged surfactant to increase the chance of drug encapsulation. The heating of the system was found to be very important during the melting and mixing of the solid lipid. Upon cooling, pre-emulsion has smaller droplets, which may result in smaller nanoparticles. It was observed that the cellular uptake and distribution of SLNs were influenced by the particle size, surface charge, type of lipids, and the concentration of the emulsion [19,20]. TEM imaging confirmed that the synthesized Res-SLNs were spherical with a narrow size distribution. The mean particle size of Res-SLNs in our study was about 168.2 ± 10.7 nm. It had been reported that the spherical particles with a size below 200 nm preferentially accumulated in tumor tissues owing to the enhanced permeability and retention (EPR) effect [21]. The polydispersity index (PDI) is a marker for the homogeneity of particle size distribution. PDI in the range from 0.15 to 0.3 indicates a good homogeneous size distribution [22]. In this study, the PDI of Res-SLNs formulations was about 0.26 which indicated that Res-SLNs exhibited a good homogeneous size distribution. Zeta potential is an important factor which obviously affects the stability drug delivery system. Usually, absolute large negative or positive zeta potential value is required for colloidal dispersion stability because electrostatic repulsion between particles with same charges can avoid aggregation [23]. A negative potential of −23.5 mV is high enough to avoid the aggregation of nanoparticles and keep their dispersion system stable. The UV–vis spectra and XRD for the Res-SLNs clearly demonstrate the chemical integrity of Res in the SLNs, which confirms that encapsulated resveratrol retains its chemical properties inside the SLNs.

In the present study, the breast cancer cell line MDA-MB-231 was selected to explore the anti-cancer effect of resveratrol and Res-SLNs. Previous publications had found that resveratrol could decrease MDA-MB-231 cell viability, arrest the cell cycle at G0/G1, and promote cell apoptosis [24]. Our present study indicated that Res and Res-SLNs inhibited cell proliferation in a dose-dependent manner, and the IC_50_ of Res-SLNs was lower than that of free Res. Blank SLNs did not show any significant toxicity to the cancer cells. As we know, the bioavailability of resveratrol is typically very low. This is due to its poor solubility in water and fast pre-systemic metabolism. Formulating resveratrol in polymeric or lipid based delivery systems (e.g., nanoparticles) is one possible strategy to overcome the drawbacks of the drug. The enhanced anti-cancer effect of Res-SLNs compared to free Res may contribute to the lipophilic nature of the carrier which facilitates the intracellular uptake. Apoptosis is one form of programmed cell death that plays a pivotal role in cancer therapy. Bcl-2, as the nodal point at the convergence of multiple pathways with broad relevance to oncology, is well known for its ability to suppress apoptosis [25]. On the contrary, Bax proteins can induce cell apoptosis. In this study, significantly increased level of Bax and decreased level of Bcl-2 were found after the treatment of res-SLNs. Furthermore, the cell cycle in the G0/G1 phase significantly increased in both Res and Res-SLNs treatment groups, compared with control groups. CyclinD1 is a cell cycle-related protein. Many studies have reported that G1/S progression is highly regulated by CyclinD1, and the loss of CyclinD1 can induce G1 phase arrest [26,27]. The downregulation of CyclinD1 was found in the Res-SLNs-treated MDA-MB-231 cells which indicates that Res-SLNs increase the cell cycle arrest in the G0/G1 phase via the mechanism of downregulation of CyclinD1 in cancer cells. 

## 4. Materials and Methods

### 4.1. Materials

Resveratrol (Res, 99%) was purchased from Aladdin (Shanghai, China). Stearic acid, lecithin chloroform, and Tween^®^ 80 were obtained from Sinopharm Chemical Reagent Co, Ltd. (Shanghai, China). The water was prepared with a Millipore (Bedford, MA, USA) Milli-Q^®^ system, and all other chemicals were analytical grade. Polyoxyethylene(40)stearate (Myrj 52) and acetonitrile (high-pressure liquid chromatography [HPLC] grade) were purchased from Sigma-Aldrich Corporation (St. Louis, MO, USA).

### 4.2. Preparation of Resveratrol-Loaded Solid Lipid Nanoparticles

Resveratrol-SLNs were prepared using the solvent injection method. Briefly, Resveratrol (150 mg), stearic acid (200 mg), and lecithin (100 mg) were dissolved in 10 mL chloroform in a glass flask (as organic phase). Myrj 52 was dissolved in 30 mL distilled water and heated to 75 °C ± 2 °C in a water bath (as aqueous phase). The organic phase was injected into the hot aqueous phase under mechanical agitation at 1000 rpm, and the resulting solution was kept at 75 °C with the same agitation speed to remove the organic solvent. The condensed solvent (approximately 5 mL) was then transferred into an equivalent amount of cold water (0–2 °C) under continuous mechanical stirring (1000 rpm) for 2 h. The resultant suspension was centrifuged at 20,000 rpm (Avanti J25 centrifuge, JA 25.50 rotor; Beckman Coulter, Palo Alto, CA, USA) to remove the supernatant. The pellet was suspended in ultrapure water, refrigerated, and lyophilized. 

### 4.3. Morphology, Size Distribution, and Surface Charge Detected by TEM and Zetasizer

A drop of diluted solution of the Res-SLNs was placed in carbon-coated copper TEM grid (150 mesh, Ted Pella Inc., Rodding, CA, USA), the samples were imaged using a JEM-1230 transmission electron microscope (JEOL, Tokyo, Japan) and visualized at 120 kV under microscope. The particle size and the zeta potential of Res-SLNs are determined at 25 °C by photon correlation spectroscopy (Zetasizer Nano ZS, Malvern Instruments, Malvern, UK).

### 4.4. Quantifying the Loading Efficiency of Res by UV–Vis Spectroscopy

The amount of entrapped Res was determined by UV–vis spectroscopy. A sample containing a determined weight of Res-SLNs was dissolved in the determined volume of ethanol. After the sample was completely dissolved, the concentration of Res was determined with a UV–vis spectrophotometer. The selected wavelength for Res measurement was 304 nm.

$$\mathrm{Drug}\;\mathrm{loading}\;\%\;=\;\frac{\mathrm{Weight}\;\mathrm{of}\;\mathrm{Res}\;\mathrm{in}\;\mathrm{Res}-\mathrm{SLNs}}{\mathrm{Weight}\;\mathrm{of}\;\mathrm{Res}-\mathrm{SLNs}}\;\times \;100\%$$

### 4.5. UV–Vis Spectra and X-ray Powder Diffraction Analysis

The UV–vis spectra (Varian, Victoria, Australia) were observed to investigate the possible chemical interactions. The absorbance spectra of free Res, Res-SLNs (dissolved in ethanol), and SLNs were measured using a Cary 50 UV–vis absorbance spectrophotometer. X-ray diffraction measurements were performed in order to characterize the crystallographic structure of the resveratrol-SLNs, resveratrol, and SLNs. The patterns were carried out with an X-ray diffractometer (D8 Advance; Bruker, Karlsruhe, Germany) in the range of 5°–50°. The measurements were performed at a voltage of 40 kV and 25 mA.

### 4.6. Cell Culture

The MDA-MB-231 (Human breast cancer) cells were obtained from ATTC. The cells were cultured in Dulbecco’s modified Eagle’s medium (DMEM; Gibco, Gaithersburg, MD, USA) supplemented with 10% fetal bovine serum and 1% antibiotic solution and maintained at 37 °C in a 5% CO_2_ incubator.

### 4.7. Cytotoxicity Study

The sulforhodamine-B (SRB) assay was performed to assess the potential antiproliferative effects of Res, Res-SLNs, and SLNs on MDA-MB-231 breast cancer cells. The cells (1 × 10^5^ per well) were seeded in 96 well plates and incubated for 24 h in a 5% CO_2_ incubator. Afterwards, media was replaced with fresh media containing different equivalent concentrations of free Res and Res-SLNs and incubated for 24 h. After incubation, the cells were fixed in ice-cold trichloroacetic acid (10% *w*/*v*) for 1 h. The cells were washed and stained 0.4% SRB dye for 30 min at room temperature. The excess SRB dye was removed and 100 μL of 10 mM Tris buffer (pH 10.5) was added. The optical density of each well was measured at 515 nm on a Bio-Rad 550 ELISA microplate reader. All experiments were performed at least three times. 

### 4.8. Hoechst 33342 Staining

MDA-MB-231 cells were seeded into 6-well plates at a cell density of 1 × 10^4^ cells per well, cultured overnight, and treated with Res and Res-SLNs for 24 h. The suspension was removed, and 1 μg/mL of Hoechst 33342 (Beyotime Institute of Biotechnology, Haimen, Jiangsu, China) was added and incubated at 37 °C for 20 min in the dark. Thereafter, the dye liquor was removed, and the cells were washed three times with PBS. A fluorescence microscope (Olympus U-RFLT50, Tokyo, Japan) was used for the examination of each well at different fields of view.

### 4.9. Apoptosis and Cell Cycle Analysis

Apoptosis were assayed using the multifunctional Muse Annexin V and Dead Cell kit (Millipore, Billerica, MA, USA) according to the user’s guide and the manufacturer’s instructions. Briefly, the breast cancer cells were harvested and washed with PBS twice. Then, 5 μL of FITC-labeled enhanced annexin V and 5 μL (20 μg/mL) of propidium iodide were added to a 100 μL cell suspension. After incubation in the dark for 15 min at room temperature, the samples were immediately analyzed by Muse Cell Analyzer (Merck, Millipore). All experiments were performed in triplicate. The cell cycle distribution analysis was measured using a Muse Cell Cycle Assay Kit (Merck-Millipore) according to the manufacturer’s instructions. Cells were trypsinized, washed with PBS and fixed in 70% ethanol overnight. Then, cells were centrifuged, washed with PBS, and analyzed by Muse Cell Analyzer (Merck, Millipore).

### 4.10. Wound Healing Assay

The breast cancer cells were seeded in 6-well plate until the cells reached to 90–95% confluency. The scratch wound was generated in the surface of the plates using a pipette tip in cells with Res, SLNs, and Res-SLNs treatment. Photographic images were taken at 0 and 24 h. The images were then analyzed using Image J software (NIH, Bethesda, MD, USA).

### 4.11. Transwell Migration and Invasion Assays

The migration of cells was conducted using a 24-well transwell chamber (Corning Incorporated, NY, USA) with gelatin-coated polycarbonate membrane filter. The invasive capacity of cells was performed using transwell precoated with matrigel (BD Biosciences, San Jose, CA, USA). After incubation for 24 h, the upper surfaces of the transwell chambers were scraped with cotton swabs, and the migrated and invaded cells were fixed with 4% paraformaldehyde, and then stained with Giemsa solution. The stained cells were photographed.

### 4.12. Western Blot Analysis

MDA-MB-231 cells were seeded in six-well plates (1 × 10^6^ per well) and treated with Res and Res-SLNs (40 μM) for 48 h. After drug treatment, cells were harvested and resuspended in lysis buffer. All the samples were then centrifuged at 12,000 rpm at 4 °C for 10 min. The protein concentrations of cell lysates were determined using the bicinchoninic acid (BCA) protein assay kit (Beyotime Institute of Biotechnology, Haimen, Jiangsu, China). Total proteins (20 μg) were subjected to 10% acrylamide SDS-PAGE and transferred to polyvinylidene difluoride (PVDF) membranes, and blocked with 5% nonfat milk in PBS with Tween 20 (PBST) at room temperature for 1 h. The membranes were probed with specific primary antibodies against Bcl-2, Bax, c-Myc, and CyclinD1 (Cell Signaling Technology, Beverly, MA, USA) overnight at 4 °C. After incubation with the secondary antibody for 1 h at 20 °C, membranes were washed five times. The immunoreactivity bands were detected by ECL detection system. Quantification of protein bands was performed using the Image J sofeware (NIH, Bethesda, MD, USA).

### 4.13. Statistical Analysis

All the data are represented as the mean ± standard derivation (SD). Statistical analysis was performed using the one-way analysis of variance (ANOVA). Values of *p* < 0.05 or 0.01 were considered statistically significant.

## 5. Conclusions

In summary, resveratrol had been successfully incorporated into solid lipid nanoparticle in this study. The optimized formulation had a mean particle size of 168.24 ± 10.73 nm with a high zeta potential. Res-loaded SLNs show a remarkable advantage over free resveratrol to induce cancer cell apoptosis and death. Besides, Res-SLNs were observed to have the ability of inhibiting cell invasion. These results indicated that the SLNs could serve as a better carrier for resveratrol to enhance its anti-cancer activity. In-depth biological and in vivo studies will be carried out in our future research.
